# Isolated Left Atrial Cardiac Tamponade Caused by Pleural Effusion

**DOI:** 10.7759/cureus.11578

**Published:** 2020-11-19

**Authors:** Misbahuddin Khaja, Yaneidy Santana, Miguel A Rodriguez Guerra, Arsalan Rehmani, Jose L Perez Lara

**Affiliations:** 1 Internal Medicine/Pulmonary Critical Care, BronxCare Health System Affiliated with Icahn School of Medicine at Mount Sinai, Bronx, USA; 2 Pulmonary Medicine, BronxCare Health System Affiliated with Icahn School of Medicine at Mount Sinai, Bronx, USA; 3 Internal Medicine, BronxCare Health System Affiliated with Icahn School of Medicine at Mount Sinai, Bronx, USA; 4 Cardiology, Bronx Lebanon Hospital Center Affiliated with Icahn School of Medicine at Mount Sinai, Bronx, USA

**Keywords:** cardiac tamponade, pleural effusion, pericardial effusion

## Abstract

A localized left atrial tamponade caused by left side pleural effusion is a rare finding that leads to hemodynamic instability. Here, we describe left atrial systolic and diastolic collapse resulting from left pleural effusion. An increase in intrapleural pressure by a pleural effusion can compress the pericardial space and lead to impaired cardiac filling and tamponade physiology. Here, we present a case of a 79-year old African American female who presented with shortness of breath and dry cough for a duration of one week. Chest radiograph and CT scan of the chest showed left pleural effusion. The echocardiogram revealed left atrial systolic and diastolic collapse due to pleural effusion, which triggered cardiac tamponade physiology. With the guidance of a bedside thoracic ultrasound, she underwent a diagnostic and therapeutic thoracentesis which resolved her symptoms. Repeat echocardiogram revealed resolution of the cardiac tamponade with no further indication of left atrial diastolic collapse. In conclusion, pleural effusions can cause tamponade physiology and can be resolved by thoracentesis. Early recognition by a bedside point-of-care ultrasound may help provide prompt relief of tamponade.

## Introduction

Cardiac tamponade is a medical emergency that is most commonly caused by pericardial effusion which compresses all cardiac chambers and raises the pericardial pressure, often resulting in hemodynamic instability. However, in rare instances, a massive pleural effusion can cause cardiac tamponade in the absence of pericardial effusion [1].

Cardiac tamponade can be acute or subacute, with varying etiology. Most patients have one of the following physical findings: sinus tachycardia, pulses paradoxus, and/or elevated jugular venous pressure [2].

A massive pleural effusion can be a medical emergency. A large right pleural effusions can elevate the intrapleural pressure and lead to subsequent elevation in intrapericardial pressure. This ultimately leads to right ventricular collapse and creates cardiac tamponade physiology. It is postulated that a left sided effusion can give rise to tamponade physiology, as most of the cardiac mass is on the left. However, pleural effusions on either side can cause similar tamponade physiology [3].

An echocardiogram should be performed when there is a high suspicion of tamponade. Classic echocardiogram findings include pericardial effusion, right ventricular diastolic collapse, right atrial systolic collapse, and the inferior vena cava with minimal respiratory variation [4].

Past reports of pleural effusions causing right ventricular diastolic collapse do exist. Our case is unique because the left-sided pleural effusion resulted in an isolated left atrial diastolic collapse and generated tamponade physiology. Large pleural effusions are associated with shortness of breath; however, in rare instances, they are also associated with cardiac tamponade [5].

## Case presentation

A 79-year-old African American female was admitted to our hospital following shortness of breath and a dry cough for one week duration. Her past medical history included chronic obstructive pulmonary disease on home oxygen, coronary artery disease, recurrent gastrointestinal bleed with duodenal arteriovenous malformation, factor VIII inhibitor, and factor IX deficiency. She had no complaint of hemoptysis, chest pain or abdomen pain, no recent travel or sick contacts. She denied using any recreational drugs but was an ex-smoker who had quit smoking the previous year. She previously smoked for nearly 50 years.

Upon physical examination, the patient was in moderate respiratory distress, afebrile with a temperature of 98°F, heart rate of 96 beats per minute, blood pressure of 74/50mm Hg, respiratory rate of 20 breaths per minute, and oxygen saturation of 90% on 4 liters of oxygen. Bronchial breathing sounds was noted on the right side of the chest, with decreased breath sounds on left side. Upon cardiovascular examination, the heart sounds were observed to be normal. The abdomen was soft upon palpation, with no organomegaly noted. Pedal edema was present on the bilateral extremities.

**Figure 1:**
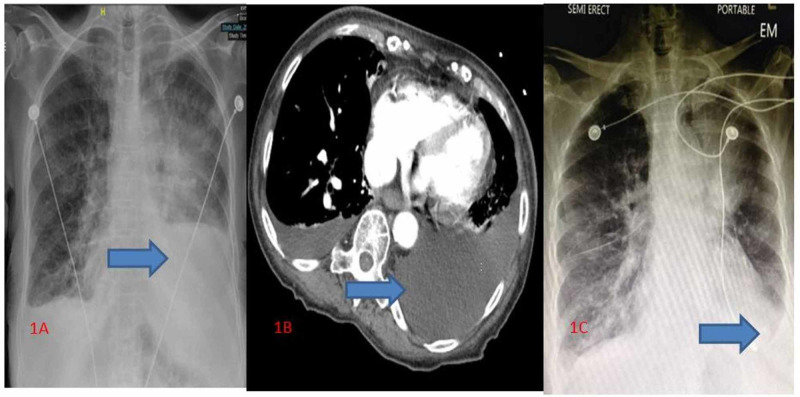
Chest X-ray, chest CT, and chest X-ray post-thoracentesis (A) Chest X-ray showed large left pleural effusion, minimal right pleural effusion, and left upper lobe infiltrate. (B) CT scan of the chest showed large left pleural effusion with minimal right pleural effusion. (C): Chest X-ray post-thoracentesis showing decrease in pleural effusion

The laboratory analysis was pertinent for leukocytosis (14 k/ul), with hemoglobin and hematocrit values of 10.8 g/dl and 33%, respectively. The comprehensive metabolic panel was within normal limits, but her lactic acid was elevated at 5 mmoles/L. A chest X-ray showed a large left pleural effusion, a minimal right pleural effusion, and left upper lobe infiltrate (Figure 1A). A Computed Tomography (CT) scan of the chest showed a large left pleural effusion with a minimal right pleural effusion (Figure 1B). A chest X-ray done post-thoracentesis showed a decrease in the left pleural effusion (Figure 1C).

**Figure 2:**
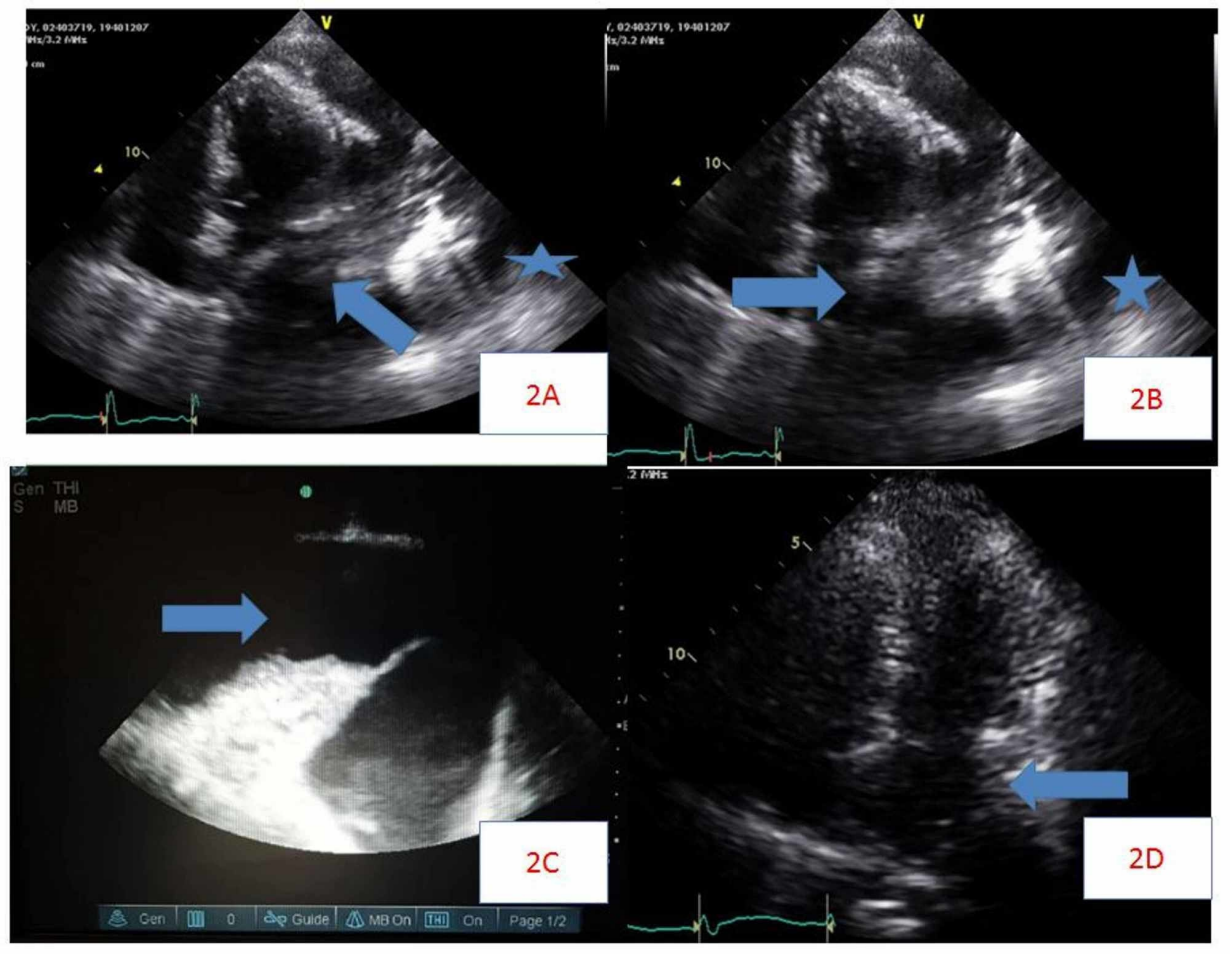
Echocardiogram findings Echocardiogram showing diastolic (A) and systolic (B) collapse of left atrium (arrow) due to pleural effusion (star) causing cardiac tamponade. Thoracic ultrasound (C) showing large left pleural effusion (arrow). Echocardiogram done after thoracentesis (D), showing no diastolic collapse of the left atrium (arrow), with resolution of cardiac tamponade

The patient was transferred to the intensive care unit, started on fluid resuscitation, oxygen supplementation with nasal canula. A bedside two-dimensional echocardiogram was done, which revealed an ejection fraction of 55%, with left atrial systolic and diastolic collapse due to a pleural effusion causing cardiac tamponade. No systolic collapse of the right atrium was observed, and the right ventricular structure and function was normal, with no right ventricular diastolic collapse. The left ventricular size and function were normal. Inferior vena cava collapse was observed with inspiration. No significant valvular abnormalities (Figure 2A, 2B) were seen. A bedside thoracic ultrasound was performed, which showed a large left pleural effusion (Figure 2C). A repeat two-dimensional echocardiogram was performed after thoracentesis, which showed no diastolic and systolic collapse of the left atrium, with resolution of cardiac tamponade (Figure 2D).

The patient underwent a diagnostic and therapeutic bedside thoracentesis by intensivist where 850 ml of serous pleural fluid was drained from the left pleural space. Analysis of the pleural fluid showed protein 2.9 g/dl, LDH 644 U/l, and glucose 269 mg/dl, with lymphocytic-predominant complicated para-pneumonic exudative effusion. Pleural fluid cultures showed no growth of organisms. Pleural fluid cytology showed highly atypical cells favoring adenocarcinoma of the lung.

After drainage of pleural effusion, her symptoms resolved. Repeat lactic acid was 0.9 mmoles/L, blood pressure improved to 112/64 mm Hg. She did not need pressor support during her resuscitation. When pleural fluid cytology was positive for malignant cells, she was referred to an oncologist. Because of her poor functional status, the patient declined chemotherapy for her newly diagnosed stage IV lung adenocarcinoma of the lung and opted for palliative care.

## Discussion

Cardiac tamponade should be included as one of the differentials in critically ill hemodynamic unstable patients. Bedside echocardiogram can aid the diagnosis and management of cardiac tamponades. The most common causes of cardiac tamponade are trauma, cardiac surgery, ventricular free wall rupture, infections, inflammatory diseases, metabolic, drugs, and malignancy. The noncardiac, extra-pericardial causes of cardiac tamponade, include pleural effusion, pneumothorax, elevated intraabdominal pressure, and positive pressure ventilation [6].

During the early phase, patients may be asymptomatic. However, upon increase in intrapericardial pressure, the patients will present with dyspnea, chest heaviness, pain, and pedal edema. Upon examination, the patients have elevated jugular venous pressure, absent Y descent, pulses paradoxus, and narrow pulse pressure with hypotension with decreased stroke volume. External compression of the myocardium by pericardial effusion results in decreased cardiac filling and hemodynamic instability [7].

Accumulation of pericardial fluid in the pericardial space leads to an increase in intrapericardial pressure, which causes equalization of diastolic pressure. As ventricular diastolic filling decreases, it causes a reduction in stroke volume. This leads to an increase in myocardial contractility and tachycardia from the compensatory mechanism of sympathetic activation. In the case of a large pleural effusion, the elevated intrapleural pressures may cause compression of the pericardium resulting in tamponade physiology with hemodynamic instability [8].

As pleural effusion accumulates, the cardiac chamber's compression leads to a decrease in cardiac output, stroke volume, mixed venous saturation, mean arterial pressure, and left ventricular preload [9]. In 1991, Kisanuki et al. first reported a malignant pleural effusion causing left ventricular diastolic collapse with no collapse of the right ventricle, right atrium, and left atrium, and normalization of the left ventricular wall after drainage of pleural effusion [10].

Sadaniantz et al. performed a retrospective analysis, after reviewing 116 echocardiograms in patients without pericardial effusion but with pleural effusion, and showed right atrial diastolic collapse in 18% of patients. However, our patient had left atrial diastolic collapse secondary to a pleural effusion [11].

The highest prevalence of collapse is seen in the right atrium because it is a low-pressure chamber with thin walls. Next is the right ventricular free wall collapse in early diastole. The longer the duration of atrial collapse, the more severe the cardiac tamponade. If the intrapericardial pressure exceeds that of the right atrium and right ventricle, both right atrial and right ventricular diastolic collapse is observed. In early tamponade, right ventricular collapse is seen during expiration because the minimum pressure falls below the pericardial pressure. But when intrapericardial pressure is increased, the right ventricular collapse is recognized throughout the respiratory cycle. In severe tamponade, sometimes a left atrial collapse with timing similar to right atrial collapse is observed [12].

In some instances, the right ventricular diastolic collapse was not observed, even when the patient had pericardial effusion, indicating right ventricular hypertrophy and a sudden increase in right heart filling pressure. On the other hand, acute mitral regurgitation due to acute ischemia may cause right ventricular diastolic collapse even in the absence of pericardial effusion.

The increase in intrapericardial pressure is better tolerated if caused by pleural effusion than pericardial effusion, as pleural effusions compress both the lungs and the thoracic vasculature. Right ventricular diastolic collapse occurs during inspiration with pericardial effusion and expiration with pleural effusion. Pericardial effusion can cause the pulmonary arterial pressure to fall below that of the left atrial blood pressure, suggesting that blood transport depends greater on the energy from respiratory activity than on right ventricular contraction [13].

Post-cardiac surgery loculation from hematoma over the right atrium can cause cardiac tamponade. Also, a loculated effusion after right ventricular infarction can cause right side tamponade [14]

Although a transthoracic echocardiogram is needed to identify cardiac tamponade, Alam et al. reported that post-cardiac surgery patients could develop tamponade despite having small to moderate effusion post-operative mediastinal changes, require a transesophageal echocardiogram to define the tamponade better [5].

Treatment of cardiac tamponade depends on several factors, including history, pericardial effusion (if loculated or circumferential), etiology of effusion, acute or chronic, whether intrapericardial pressure is elevated, and whether the patient needs open drainage or pericardiocentesis. Patients with hemodynamic instability with pericardial effusion need urgent drainage for both diagnostic and therapeutic purposes. Patients who are hemodynamically stable with pericardial effusion do not require immediate drainage but will eventually require diagnostic pericardiocentesis if malignancy is suspected [15].

Pericardiocentesis is the most common method to drain pericardial fluid via echocardiographic or fluoroscopic guidance, and an indwelling percutaneous catheter can be left in the pericardial space [16].

Our patient had a large pleural effusion causing cardiac tamponade physiology, which can be relieved by thoracentesis. A bedside lung ultrasound can help in identifying pleural effusion. A moderate to large pleural effusion causing tamponade physiology needs immediate thoracentesis rather than volume resuscitation, pericardiocentesis, or inotropic agents, which are the typical treatments for pericardial effusion [17].

## Conclusions

Our case report describes a case of isolated left atrial cardiac tamponade physiology caused by a large left pleural effusion. However, there have been reports in the past where the right atrium and right ventricle have presented with cardiac tamponade physiology due to pleural effusion. In the present case, symptoms resolved immediately after thoracentesis, underscoring bedside ultrasound utility in critically ill patients. An intensivist with knowledge of bedside point-of-care ultrasound echocardiogram can recognize early tamponade physiology due to pleural effusion. In that case, an immediate thoracentesis may lead to symptomatic relief. Our case presents a rare occurrence and reminder of left side pleural effusion causing isolated left atrial tamponade.
